# Translation Notes: Heidegger, Derrida, and the Chance for (a) Philosophy

**DOI:** 10.12688/openreseurope.17093.2

**Published:** 2024-05-28

**Authors:** Elena Nardelli

**Affiliations:** 1 Universita degli Studi di Padova, Padua, Veneto, Italy

**Keywords:** Philosophy of Translation, Philosophical Praxis, Deconstruction, Original, Translation

## Abstract

This paper aims to show that translation is not only a fully-fledged philosophical problem, but also a specific philosophical praxis and a test bed for extracting the core of different philosophical frameworks. For this purpose, I will take into consideration the respective philosophies of Martin Heidegger and Jacques Derrida. Even if Heidegger often practices translation from the Greek in his own works and adds a few remarks towards an original investigation of this activity, he ultimately understands translation as a ‘makeshift’ or as a ‘shipwreck’. Throughout his contestation of Heidegger’s position, Derrida shows the trap of the endless appropriation of the experience of the origin structure. He also frees up the discourse by putting the hierarchical polarization between the original and the translation into question. Thus, translation becomes a chance for philosophy, even for Derrida’s deconstruction, a chance to generate new paths for investigation and to keep its question open.

## Heidegger’s notes on translation

Although Heidegger does not explicitly dedicate any essay to the subject of translation, it is difficult to find a volume of the *Gesamtausgabe* in which Heidegger does not actually translate something. And when he does, he leaves hints of theoretical reflections on this activity in annotations, comments, and observations; the richest of these are scattered throughout the materials for the courses he gave in Freiburg during the first half of the 1940’s (for example, in *Hölderlin’s Hymn ‘The Ister’* [SoSe 1942], *Parmenides* [WiSe 1942/43], and *Heraclitus* [SoSe 1943]) or in the writings just afterwards – that is, *Anaximander’s Saying* (1946) and *Off the Beaten Track* (1950), *The Principle of Reason* (1955/56) and *On the Way To Language* (1950–1959). In accordance with these texts, the concept of translation turns out to be at the crossroads of the main *Grundfragen* of Heideggerian reflection: the question of language, the relationship between the said and unsaid, the remembering and appropriation of a path towards the origin, and the reworking of the Western philosophical tradition. I will try to show in this paper that translation is not only a fully-fledged philosophical problem and an immanent element of philosophical praxis, but that it is also a tool we can use to question specific philosophical positions, in this case those of Martin Heidegger and Jacques Derrida, and to inquire into their intimate and firmest convictions. In this regard, the paper aims to demonstrate the validity of Heidegger’s laconic intuition: ‘Tell me what you think of translation, and I will tell you who you are’ ( Heidegger, [1984] 1996, 63).

The most common instances of translation in Heidegger’s discourse occurs at moments like this: he is giving a lesson (for example, on Parmenides or Heraclitus); he reads a passage in Greek and then proposes his own German translation of it. At this point, Heidegger seems to suddenly realize that this is not an innocuous or obvious activity, but one that requires a certain amount of justification. This justification, however, is perceived as a pause interrupting the thread of the discourse and is therefore inserted between brackets and/or quotation marks and/or is labeled *Zwischenbemerkung* – that is, as passing or accidental observation. Here Heidegger formulates his idea on translation as such, in reflections that are not homogeneous and consequently vary from work to work; sometimes translation is a necessary evil, *Notbehelf* and *Irrfahrt*, whereas elsewhere it ‘concerns the relation of human beings to the essence of the word and to the worthiness of language’ ( Heidegger, [1984] 1996, 63). At times, as in the course on Parmenides, when Heidegger seems to come very close to a decisive point in understanding the activity of translation, the discussion is deferred to a later time. I will provide a general overview of Heidegger’s comments on translation by pointing out five constant elements that characterize Heidegger’s work on translation. From these first signs, we see a clear difference between the way we usually think about translation as a regulated equivalence between two stand-alone linguistic systems.

a.) First, the terms ‘translation ( *Übersetzung*)’ and ‘to translate ( *übersetzen*)’ often appear in quotation marks. This is symptomatic of Heidegger’s particular caution when using these terms, which signals that a problem has been identified and that distance is required from the conventional use of the terms; thus indicating the complexity of the question raised and inviting a kind of monitoring of the same.

b.) Second, Heidegger often refers to translation with the binomial ‘denkende Übersetzung,’ thinking translation, which suggests a qualitative distinction between the commonly understood translation process and Heidegger’s ‘thinking’ ( *denkende*) reformulation.

c.) Third, the act of translation is described as a jump over a ditch ( *der Sprung über einen Graben*). Those familiar with Heidegger's thought know that jumping is a pivotal act in his work, especially for the component of tension towards the ‘other beginning’. What interests me here is that the greatest obstacle to translation, the gap that we must overcome with a leap, seems to be created by our ‘natural’ relationship with language. And therefore, somewhat paradoxically, in order to enter into the passage that will be translated it is necessary to leap out of it and escape the usual interpretation conveyed by conventional language. Thus the act of translation is described as a simultaneous movement of distancing and approaching.

d.) Fourth, Heidegger awakens and exploits the second meaning of the German term ‘übersetzen.’ When the accent is on the word *setzen*, it means to translate; but when the accent is on *über*, it means to cross to another shore. And Heidegger points to this second, ‘hidden,’ meaning as the more authentic one.

e.) Fifth, translation often appears in Heidegger’s invention of the reflexive verb ‘to translate oneself ( *sich übersetzten*).’ What is implied in the shift from the transitive to the reflexive form of the verb? It seems to be a move away from the usual understanding of translation as the operation of a subject moving an object from one linguistic system to another. This shift certainly implies the abandonment of every (meta-)position of exteriority to the translation process that would allow measured transpositions between comparable languages. The result is therefore something similar to the way in which Derrida describes the middle voice of the verb in *La différance:* ‘an operation that is not an operation, which does not allow itself to be considered either as an action or as a passion of a subject on an object whether starting from an agent or starting from a patient’ ( Derrida, [1972] 1982, 9). Furthermore, the reflexivity of translating discloses the dimension of the task as a work about ourselves, activating the transformative dimension of existence by disturbing everyday life and habits. The overall effect of this is estrangement, where the usual and the familiar cease to be perceived as such and thereby manifest the presuppositions on which they rest. This is what Heidegger asks of his students; he asks to revive the sense of philosophical digging into the texts that have been handed down to us: to translate ourselves means to forge a dialogue with the thinkers of the past and to bring the questions that arise from this dialogue into our existence. Thus translation is the way to live a question, reiterate it, and hazard a response. But if the reflexivity of translation seems to solicit a change of place on our part, in what direction should we look? Where are we heading when we translate ourselves?

Heidegger ([1950] 2002, 255–256) writes in *Anaximander’s Saying*:

For what is necessary before interpreting the saying is to trans-late ourselves – at first without the saying – to the place from which what is said in the saying comes; to, that is to say, τὰ ὄντα. This word names that of which the saying speaks, not only what it expresses. That of which it speaks is already, before its expression, what is spoken about by the Greek language in both its everyday and its elevated use. For this reason we must seek the opportunity which allows us to trans-late ourselves outside the saying itself, in order to discover what τὰ ὄντα, thought in the Greek way, says.

Here Heidegger seems to urge us to translate ourselves ‘outside of the fragment,’ and into the Greek experience – that is, to try to appropriate that particular Greek modality of the being’s manifestation. In other words, we should acquire the Greek modality in which human beings say something about the world, say something about their relationship with the world, and say something about the being of beings as it comes to the word. This Greek experience, however, is not available in a ready-to-wear form; it must be conquered through the act of translation. The words we have received as an inheritance, those texts that have come down to us (such as the fragments of Anaximander, Parmenides or Heraclitus), serve us as a riverbank; they are a point of ricochet, retrospectively indicating the original experience Heidegger aims to gain. And those words have no existence in themselves; they have no fundamental given meaning, no pure state that deteriorates with the passage of time or is watered down as it moves further away from its purity. This is because the fundamental meaning of a word is something that manifests itself only at the end of a very long journey – that is, the journey of this term throughout the Western tradition, in its transmission and in its passage through the various ages. And translation opens the way, as suggest Parvis Emad, ‘originary translation occurs as way-making/saying/showing’ ( 1993, 330).

However, to reveal oneself only *a posteriori* requires someone to listen to what is said, and to inherit it in the full sense of the term; it is to provide a place for it to resonate with oneself without allowing it to echo. The unsaid would remain unapproachable and ineffable if no one was disposed to make it resonate. Following Jean-Luc Nancy’s suggestion ( [2002] 2007), we can think about the translator’s subjectivity as a diapason. The activity of translation is therefore intrinsically a secondary and supplementary activity, but this secondary and supplemental nature is not a disqualification; on the contrary, it serves as a resource.

When we consider the Greek word as the foreign word to be translated, it is not foreign in the sense that it is external to us, to our tradition; it is the foreign within us, a previously unthought of origin. And the work to be done, to immerse oneself in the experience of this word, is a version of anamnesis. The translation acts as a remembrance, the way a thought reminds us of something, as Heidegger says, it is an *Andenken*. It is Hölderlin’s poetry that enables us to envision this path of a river flowing upwards in the direction of its spring. Our origin is the most difficult thing to make our own, and thus our translation is a translation into ourselves, towards our source with the appropriation of our ownmost perspective.

In this sense, translating is as much an *Andenken* (a remembrance) as it is an *Aneignen* (an appropriation). Since everyone is affected by the presbyopia of their own era, according to Heidegger, everyone misses their own experience. One’s own, the original, is not a ‘given’; it is not something that belongs to us by right or by default, but it is that which is most foreign to us. And translation is an attempt to conquer this, or at least to grasp this origin posthumously and retrospectively. Heidegger points out that even our own language is not something we possess ‘naturally’ or spontaneously at birth; it is something we have to continually learn, because it is the most difficult and it is rarely mastered. And ultimately, this is why translation takes place primarily within our own language.

To sum up, Heidegger understands translation in a reflexive way where the inclination in the direction of the foreign (which for Heidegger is not French or Japanese, but always Greek) coincides with an inclination towards the stranger in ourselves, towards ‘the primal have-been’ with a view of returning home.

## Before translation’s mirror

I will now try to retrace a few aspects of Jacques Derrida’s confrontation with his Heideggerian inheritance, especially with respect to the concept of origin/original. With this study, I hope to clarify the different logical-ontological structures that come to light during an inquiry into translation. As I have mentioned earlier, for Heidegger the so-called original text, the text to be translated, serves as a point of ricochet or compass – it is not the ultimate goal of the translational activity. What is of interest to Heidegger is to approach the experiential horizon that this word preserves in itself, to bring to light what he believes has remained unthought, but is still indicated in an indirect way – that is, the origin of Western thought. This origin seems to be intrinsically ambiguous and inexhaustible, constantly reconfigured but never fully or definitively stated. And this is the reason why Heidegger never ceases to propose new translations for these original words over the course of his intellectual path – for example, he translates ἀλήθεια sometimes as ‘Unverhüllung,’ or as ‘Unverborgenheit,’ or as ‘Entbergung.’

Derrida places the notion of origin within a family of concepts that center around the Latin word ‘pater,’ which includes ‘head,’ ‘capital,’ and the ‘good’: this is the ‘family’ schematic paradigm. When commenting on passage 515c from the *Republic*, Derrida identifies the concept of the structural impossibility of expressing the origin as one of the constants of philosophical inquiry from Plato through Heidegger:

Now, about this father, this capital, this good, this origin of value and of appearing beings, it is not possible to speak simply or directly. First of all because it is no more possible to look them in the face than to stare at the sun. […] And since one cannot speak of that which enables one to speak (being forbidden to speak of it or to speak to it face to face), one will speak only of that which speaks and of things that, with a single exception, one is constantly speaking of ( Derrida, [1972] 1981, 82–83).

In this sequence, Derrida also includes Heidegger’s invitation to listen to the Greek word for a possible disclosure, for a manifestation of the origin in and by itself. Derrida distances himself from this dynamic through this notion of ‘supplement.’ Firstly, a ‘supplement’ is a substitute for someone or something and highlights an absence when it takes the place of another. Secondly, the supplement is supplementary: it follows the original, arrives late, and is added afterwards. The relationship between the original and the translation, just like the relationship between origin and supplement, is not an oppositional relationship; it is a relationship of substitution, repetition, (ex)change and imitation. Furthermore, this notion of supplement Derrida developed is permeated with a paradoxical logic: the derivative precedes what follows it, it is older than what it derives from, ‘it is a non-origin which is originary’ ( Derrida, [1967] 1978, 255). But we saw this structure in action in Heidegger's translations, where his work on a Greek word tends to bring out its most original meaning that can only be created posthumously. But this is not Derrida’s stopping point. Where for Heidegger the difference between origin and supplement is clear and insuperable, for Derrida it is the structure of the supplement that calls this difference into question. In fact, in its first meaning, the supplement appears to take the place of the original and becomes confused with it as it passes through it. In a nutshell, this means an erosion of the very distinctiveness held between original and translation.

The overlap of an original text and its translation with the supposition of their indistinguishability would be considered unacceptable by not only Heidegger but also, for example, by someone like Walter Benjamin; for them, the argument regarding the difference between an original text and its translation is exemplified in the diverse translatability of the original text and the translation. According to Benjamin, an original text is always translatable, and the fact that an adequate translator may not be found in no way invalidates the fact that the text is translatable. For Benjamin, the translatability of the text is apodictic; but, at the same time, it is impossible to translate a translation: ‘translations, on the other hand, prove to be untranslatable not because of any inherent difficulty, but because of the looseness with which meaning ( *Sinn*) attaches to them’ ( Benjamin, [1923] 1969, 81). The clear demarcation between the original and the translation is possible due to Benjamin’s assumption of the original text’s essential core, something intact and intangible that maintains a natural relationship with its language. The original is no longer being transformed once it has been translated, because the language of the translation is too powerful, directed towards ‘pure language’ and is therefore unsuitable for the content which it artificially inherits and wears as a royal robe ( Benjamin, [1923] 1969, 75).

Furthermore, Derrida’s logic of substitution is self-replicating. In other words, the supplement tends to generate other supplements which in turn create more supplements, or a substitute for the substitute of the substitute, and so on. The first term in this self-replicating chain is not only lost, but is superfluous. And thus that origin Heidegger had tried to excavate in order to capture in its disruptive possibility, free of misleading subsequent stratifications, loses its privileged position. It simply becomes one textual place among others, a link in a chain of supplements sufficiently equal to, and sufficiently different from, their ‘original’ (now in quotation marks). An original word by Anaximander or Heraclitus would therefore already be supplementary in the sense that it is already (in) translation. As underlined by Andrew Benjamin what is here at stake is ‘the possibility of thinking both semantics and translation independently of there being an origin to be recovered or retrieved’ ( Benjamin, 1989, 179).

If Heidegger’s retrospective remembrance aims at re-appropriation, asking translation for a return to oneself (albeit a non-predetermined self) for Derrida to translate, is precisely the activity that demonstrates the defeat of every attempt at an identity claim. And Derrida’s attitude towards this defeat is one of contentment based on a decisive assumption – that is, that the return to oneself is not possible and nor is it desirable as the complete appropriation of anything. This is the deepest divide between Heidegger and Derrida. 

To approach this question from another direction, Derrida shatters the origin in its ambiguity and irreducibility by thinking about it in a radically plural way where every word inherits the characteristics of the origin – thus every single word is understood to be intrinsically ambiguous and irreducible – without, however, inheriting its prerogative. It is an anti-hierarchical gesture that attacks the privilege of what Heidegger calls ‘radical words’.

There will be no unique name, even if it were the name of Being. And we must think this without *nostalgia*, that is, outside of the myth of purely maternal or paternal language, a lost native country of thought. On the contrary, we must *affirm* this, in the sense in which Nietzsche puts affirmation into play, in a certain laughter and a certain step of the dance.
  From the vantage of this laughter and this dance, from the vantage of this affirmation foreign to all dialectics, the other side of nostalgia, what I will call Heideggerian *hope*, comes into question. I am not unaware how shocking this word might seem here. Nevertheless I am venturing it, without excluding any of its implications, and I relate it to what still seems to me to be the metaphysical part of ‘The Anaximander Fragment’: the quest for the proper word and the unique name ( Derrida, [1972] 1982, 27).

For Heidegger, some terms are more original than others (for example, the terms φύσις, λόγος, ἀλήθεια, χρεών as employed by the first Greek thinkers) and encompass an authentically ‘other’ experience of thought and as Escoubas has shown Heidegger’s understanding of translation ‘eliminates the theory of the sign and initiates a theory of the name’ ( 1993, 347). For Derrida every single word is already translated, and is therefore just as secondary, retrospective, etc., as it is original (if ‘original’ is understood following Heidegger’s formulation of what is intrinsically ambiguous, inexhaustible, and unpronounceable in an adequate or definitive manner and thus in need of translation). For this reason, every text is a source text just as much as it is a target text. Derrida also distrusts our attachment to names in general, because names seem to imply the desire for an appropriately irreducible and conclusive word to be understood (since Aristotle) as a non-temporal unity in contrast to a verb. This mistrust also applies to the terms that Derrida coins (which are relatively few in comparison with Heidegger) and to those which become recurrent concepts in his philosophical discourse such as ‘différance,’ ‘marge,’ ‘trace,’ or even ‘supplement’ (despite his best effort for this not to be the case). Derrida attempts to avoid focusing on the single name or the single word; instead, he tries to grasp language as a whole as a system of references and functions of substitutions.

To briefly summarize the argument, although Heidegger may assert that translation is an odyssey that always ends with a ‘shipwreck,’ and that every translation is a ‘makeshift’ and ‘poor’ to some degree ( Heidegger, [1979] 2018, 37–38), this is because he presupposes (or at least hopes) that it is possible to find another way to access a word without translation following a path that allows us to pause in the experience of the original. Therefore, Heidegger remains stuck in what John Sallis has called ‘The Dream of Nontranslation’ ( 2002, 1–20). Derrida, however, has a completely different attitude towards translation; on several occasions, as we will see, he welcomes it as a chance, or rather *the* chance for thought and for philosophy. Nevertheless, the topic of translation plays a quite marginal role in his works with the exception of *Des tours de Babel* and *What is a ‘relevant’ translation?* Derrida often touches upon the complexity and the philosophical dignity of the topic, but he severs the path for further inquiry or disquisition. On this occasion, I am not going to retrace the role translation plays in the Derridean constellation of paradoxical law, gift, economies, and metaphor. Instead, I will conclude by considering translation as the chance for philosophical thinking.

## *Déconstruction*: looking for a more beautiful word

Albeit his philosophy was and is still identified with this notion, Derrida is suspicious of the word ‘déconstruction.’ He describes it as both new and old, as an unsatisfying word to some extent, and warns against its selection or isolation. Derrida writes to his Japanese friend Izutsu: ‘I do not think that it is a good word [ *un bon mot*]. It is certainly not elegant [ *beau*]’ ( Derrida, [1987] 2008, 6). At first glance, the relationship between translation and *déconstruction* is twofold. Firstly, it enters into Derrida’s vocabulary via translation: *déconstruction* is a translation of the German word *Destruktion* as understood by Heidegger in §6 of *Being and Time*. Despite the distance between their respective philosophies, Derrida highlights his debt to Heidegger with regard to this translational moment at the core of *déconstruction*: ‘among other things I wished to translate and adapt to my own ends the Heideggerian words *Destruktion* or *Abbau*’ ( Derrida, [1987] 2008, 2). In Derrida’s early works on Heidegger, it can be noted that there is an early translational oscillation in his different solutions – for example, *destruction*, *dé-structuration*, *ébranlement*, *sollicitation* ( Derrida, 2013, 34 and 263), which eventually becomes *déconstruction*. Secondly, the French word *déconstruction* also includes an explicit reference to the process of translation. Derrida ([1987] 2008, 299) gleans it from the *Bescherelle* Dictionary:

The displacement to which the words that make up a written sentence are subjected in a foreign language, by violating, it is true, the syntax of that language, but in order to bring it closer to the syntax of the mother tongue and thus better to grasp the meaning of the words in the sentence. […] there is *deconstruction* in relation to the language of the translated author and *construction* in relation to the language of the translator.

*Déconstruction* appears then as a process of translation, which aims directly at the discourse building structures and not single lexical units. Moreover, Derrida’s understanding of translation is not based on a single word that makes up the translation atom, the *quantum* the translator works with. If translation helps us to understand what *déconstruction* is or could be and, inversely, *déconstruction* can show us what is at stake in the translation process, then we have to assume that both translation and *déconstruction* ‘share the same stakes’ ( Davis, 2001), both work with the structures of thought – that is, the reproductions and transformations of these structures, and the connections and hierarchical order between concepts. ^1^

In this way, Derrida looks at translation as a resource. At the same time, however, he is suspicious of *déconstruction* because of its reluctance to be erased due to its inclination toward ossification into a method and sequence of procedures. For this reason, Derrida ([1987] 2008, 6) makes a plea for translation by entrusting it with the concept most identified with his thought.

I do not think that translation is a secondary and derived event in relation to an original language or text. And, as I have just said, ‘deconstruction’ is a word that is essentially replaceable in a chain of substitutions. This can also be done from one language to another. The chance for (a) ‘deconstruction’ would be that another word (the same word and an other) be *found* or *invented* in Japanese to say the same thing (the same and an other), to speak of deconstruction, and to lead it elsewhere, to its being written and transcribed. In a word that will also be more beautiful.

Derrida appeals to his Japanese friend for a translation of his words and discourse, of (the) *déconstruction*. For Derrida, this means to erase and conserve the word by giving it time – that is, a time to survive or ‘Life Death’ (to use the title of Derrida’s seminar at the EHESS in 1975–76 [ Derrida, 2019]). The relation between ‘original’ and ‘translation’ should then be considered as a vital relation, as a natural relation of intimacy, where – if Benjamin is right – the life of an artwork or the life of a text is not a metaphor ( Benjamin, [1923] 1969, 71). In fact, this relation seems to understand life beyond the contraposition of organic and inorganic. To investigate life through the lens of translation also means to explore the nuances of life – for example, through the implication and contraposition of the terms ‘continued life’ ( *Fortleben)* and ‘survival’ ( *Überleben)* with death. At the same time, the so-called original appears as an organism in continuous transformation. And since the so-called original is not a fixed, self-sufficient ‘thing,’ it requires translation in the same way that lungs need air. In this sense, the translator’s commitment, much like Derrida’s request before his Japanese friend, is an attempt to answer an appeal. The translator, to be understood after Foran as a ‘sur-viving translating’ subjectivity ( 2016, 257–260), takes the responsibility for the survival of something that comes from the outside and is not properly self-sufficient.

And just as he asks for translation, Derrida attempts to undertake the translator’s task. He often makes the declaration ‘I translate’ when focusing on the topic of translation. And he makes this acknowledgement not only in reference to his translations into French – for example, of the Hegelian term ‘aufheben’ ( Derrida, [1972] 1982, 69–108) or Shakespearian verses ( Derrida, 2001) – but he also remarks on his own philosophical activity before the texts from the philosophical tradition: ‘I translate the translation by Maurice de Gandillac of a text by Benjamin who, prefacing a translation, takes it as a pretext to say to what and in what way every translator is committed’ ( Derrida, [1987] 2008, 207).

First of all, the translational attitude means that one must assume a certain distance from the purely theoretical attitude of philosophical discourse. If one considers the object of inquiry – for example, *the* beautiful, *the* human, *the* language – as a unitary core from where a polysemy of meanings spreads out, then to understand philosophy as translation may preserve the particular case being investigated (and translated) from a surreptitious universalization. If preservation is almost impossible, then alternatively the activity of translating becomes the experience of the resistance of the particular; and every translator knows the force that must be inflicted on the concrete singularity being worked on, and the remainders of that activity. The same is also valid in a recursive sense for every translation theory that tries to define what translation is in general, or what happens in the translation process as such by questioning its possibility. Furthermore, a translational element seems to be at the very core of (every) theorization, and is not only the activity of identifying risky equivalences between languages, texts, and historical moments (for example, this is what I am presently doing [in English] while working out the peculiarities of Heidegger’s, Benjamin’s and Derrida’s philosophical experiences of translation). This translation element also constitutes the permanent and immanent underbelly of the limits of theorization itself – that is, its legitimacy and implicit assumptions – and points to what is inadvertently lost. What for every translator is just poor evidence and frustration – that is, the resistance of the particular to be translated – may serve as a mirror for a moment of self-truth in every theorization.

To investigate this in more detail, understanding translation as philosophical praxis seems to be the alternative, or one of the alternatives, to apophantic discourse. The activity of translation eludes the direct assignment of a truth value. It produces situations and operates by transforming the language and the context in which it takes place. For this reason, the statement of the translator, just as Derrida’s statement ‘I translate,’ can be read as a performative utterance ^2^. This kind of utterance is part of an action where the content is not properly understood as content, but as a movement, an operation, or production. And this, as we saw, implies a commitment on behalf of the speaker/translator/philosopher (who takes charge of the violence of translation), to answer the text’s plea and take responsibility for its survival. Akin to what happens in the model of the performative utterance – for example, the marriage formula – the translator ends up in a bind, which Derrida would call, borrowing Bateson’s terminology, a ‘double bind’: the commitment to an impossible but necessary task because, in the end, nothing is untranslatable and, at the same time, nothing is translatable.

To return to Derrida’s claims, another important aspect is the exemplification of the supplement’s structure mentioned above. Derrida presents his activity as the translation of a text that is already a translation – that is, a translation of a translation. This means that the author of the so-called original text would be a translator *a priori*, bound by a previous text that asked for translation. And to prove this theoretical thesis (that every translation is translatable and that every text has a translational nature), Derrida practices translation and thereby shows this possibility. In the framework of his objection to the distinction between original and translation, Derrida touches upon the implication of this conceptual structure in the field of law. He aims to show, especially with respect to the legislation of literary property, the contradictions French jurists run into regarding translation as intellectual property and inconsistencies before the terminology involved ( Derrida, [1987] 2008, 218–221).

But Derrida ends his inquiry at this point. He does not take the following into consideration: the everyday problems that face professional translators, the role of translators in the cultural industry, or the necessity to develop a critique of translation that is both aesthetic and political in nature. This may be the task we can venture into. Derrida can follow this path of investigation until the unavoidable point where translation reveals itself to be a non-neutral activity that has a philosophical nature with a deceptive transparency. The point in question is to rethink the very concept of translation, to distance ourselves from what we have inherited, and to envision translation as a process that maintains and discloses differences. And this is possible only by questioning – with Derrida – what happens in every single particular translation, free from the desire of an ideal world without translation, without the interference of linguistic difference and the matter of language, a world without remainders and without the vertigo of the immeasurable.

## Data Availability

No data are associated with this article.

## Footnotes

^1^ For an analysis of the interplay of philosophy and translation as it occours in different philosophical traditions and periods see Benjamin (1989).

^2^ Derrida’s reading of J. L. Austin’s theory of performative utterances ( Derrida, [1972] 1982, 321–328) aims to free it from what for Derrida determines to be the dimension of the presence – that is, from the stress on intention as the center of the performative utterance organized around a teleological structured conscience.
