# Supplementary material for: Translation Notes: Heidegger, Derrida, and the Chance for (a) Philosophy
Source: Open Res Eur. 2024 May 28;4:25. Originally published 2024 Feb 12. [Version 2] doi: 10.12688/openreseurope.17093.2 (PMC11162517; doi:10.12688/openreseurope.17093.2)
Supplement: Peer review history

# Reviewer report: Reviewer response for version 2

**DOI:** 10.21956/openreseurope.19221.r47192

**Published:** 2024-12-18

**Authors:** Douglas Robinson

**Affiliations:** 1 The Chinese University of Hong Kong, Hong Kong, Hong Kong

I have to say that I have never had so little to say about an article I’ve been asked to review. This article is so good—so densely and persuasively argued and so thoroughly and complexly grounded in the textual evidence for its claims—that I kept looking in vain for something I might question, challenge, even tweak just slightly.

 I did find one passing and fairly minor (and arguably problematic) point: in summarizing Derrida’s argument in *Psyche: Inventions of the Other* the author writes:

 But Derrida ends his inquiry at this point. He does not take the following into consideration: the everyday problems that face professional translators, the role of translators in the cultural industry, or the necessity to develop a critique of translation that is both aesthetic and political in nature. This may be the task we can venture into.

 That claim, that Derrida “does not take … the everyday problems that face professional translators,” is unquestionably true of *Psyche*. But “This may be the task we can venture into” seems to be implying that he doesn’t do it anywhere. This is arguably false. The author mentions “What is a ‘Relevant’ Translation?” in passing, as one of the two articles where Derrida deals in great detail and at substantial length with translation; but it’s not clear the author has wrangled with it.

   I use the adverbs “arguably” and “problematically” because Derrida’s “relevant translation” talk/essay is a bit of a mess. As Kathryn Batchelor (2021) has shown, fully two-thirds of it were taken almost verbatim from Derrida’s seminar on mercy and forgiveness; the comments on translation that frame that discussion are desultory and somewhat hastily tacked on. But more attentive readings have come to conclusions that may be questionable—they tend to gather scattered hints and impose speculative conclusions on them—but do suggest that Derrida may have been addressing the points that the author of “Heidegger’s Notes on Translation” might be read as saying Derrida nowhere addressed.

   Michael Levine (2022: 13), for example, reads the “relevant translation” talk like this:

 That his own translation of *Aufhebung* as *relève* has proven to be “relevant” and “a good translation” no doubt bothers Derrida—“bothers” him in the sense of provoking, irritating, and unsettling him, which is to say it fascinates him. What also seems to bother him is the implicit connection he notices between a translation deemed “relevant” and an “economy” of adaptation, success, validity, adequate compensation, debt payment, the possible, and the most possible.

 That would address something like “the everyday problems that face professional translators, the role of translators in the cultural industry”—Derrida’s audience at Arles were literary translators—but the textual evidence for Levine’s reading is shaky.

 Michelle Bolduc (2023: 449) reads the “relevant translation” essay like this: “Derrida presents a rhetorical ethos that embeds translation, relevance, and mercy in a personally-inflected public reflection on the ‘Jewish Question’ and its very real historical consequences” (449). That would obviously “develop a critique of translation that is both aesthetic and political in nature.” I think the textual evidence for Bolduc’s reading is stronger, but the politics of “the Jewish Question” in Derrida’s essay is more tonal than verbal.

 So let me say that the article should definitely be published. And if the author were to say “I don’t want to change anything, I don’t believe Robinson’s passing critique is worth addressing,” and the editors were to agree, I would not protest in the slightest. As I say, it’s a problematic critique. I would also feel entirely comfortable with a footnote that cited Batchelor, Levine, and Bolduc as a critical counterpart to the claim in the text that “Derrida ends his inquiry at this point”—ends his inquiry without discussing professional translators’ everyday problems in *Psyche*—but arguably addresses something like those issues in “What is a ‘Relevant’ Translation?” I also assume that the author is the kind of inquiring mind who is going to go reread “What is a ‘Relevant’ Translation?” and maybe Batchelor, Levine, and Bolduc as well, just because it’s an interesting problem—not because there is necessarily room in this particular article to address that problem. (Full disclosure: I have been working on the “relevant” essay and the result is a 19,000-word article, or short monograph.)

Is the argument persuasive and supported by appropriate evidence?

Yes

Is the work clearly and cogently presented?

Yes

Is the topic of the essay discussed accurately in the context of the current literature?

Yes

Does the essay contribute to the cultural, historical, social understanding of the field?

Yes

Reviewer Expertise:

NA

I confirm that I have read this submission and believe that I have an appropriate level of expertise to confirm that it is of an acceptable scientific standard.

## References

[1] : Re-reading Jacques Derrida’s ‘Qu’est-ce qu’une traduction “relevante”?’ (What is a ‘relevant’ translation?). *The Translator* .2023;29(1) : 10.1080/13556509.2021.2004686 1-16 10.1080/13556509.2021.2004686

[2] : The relevance of Derrida’s translation: Mercy andethos. *The Translator* .2023;29(4) : 10.1080/13556509.2023.2249164 449-463 10.1080/13556509.2023.2249164

[3] : Translation, Transference, Trouvaille : Derrida’s “what is a ‘Relevant’ Translation?”. *Diacritics* .2022;50(3) : 10.1353/dia.2022.a908406 4-28 10.1353/dia.2022.a908406

# Reviewer report: Reviewer response for version 2

**DOI:** 10.21956/openreseurope.19221.r47183

**Published:** 2024-11-28

**Authors:** Richard Polt

**Affiliations:** 1 Xavier University, Cincinnati, Ohio, USA

The article clearly explains the resemblances and differences between Heidegger's and Derrida's approaches to translation. It ends by suggesting some more concrete philosophical research on the practice of translation.

 I have only a few small criticisms:

 In footnote 2, "presence" would be smoother English than "the presence," and by "conscience" the author probably means "consciousness" (English makes a verbal distinction between the two concepts, while Italian and other Romance languages do not).

 I am not aware of any text where Heidegger uses the word "Unverhüllung." I believe the author means "Enthüllung."

 I am not clear on the meaning of the double metaphor when the author writes that inherited texts "serve us as a riverbank; they are a point of ricochet."

 I think it would be interesting to add some quotations from Heidegger's Parmenides lectures, which include striking statements on translation. I also recommend Paul Ricoeur's brief book Sur la traduction (translated into English as On Translation), which has interesting parallels to Sallis' book by the same title. These are recommendations, not requirements.

Is the argument persuasive and supported by appropriate evidence?

Yes

Is the work clearly and cogently presented?

Yes

Is the topic of the essay discussed accurately in the context of the current literature?

Yes

Does the essay contribute to the cultural, historical, social understanding of the field?

Yes

Reviewer Expertise:

Heidegger; history of philosophy, especially ancient and 19th-20th-century continental.

I confirm that I have read this submission and believe that I have an appropriate level of expertise to confirm that it is of an acceptable scientific standard.

# Reviewer report: Reviewer response for version 2

**DOI:** 10.21956/openreseurope.19221.r41015

**Published:** 2024-06-08

**Authors:** Mette Lebech

**Affiliations:** 1 Maynooth University, Maynooth, County Kildare, Ireland

The article can be approved now

Is the argument persuasive and supported by appropriate evidence?

Yes

Is the work clearly and cogently presented?

Yes

Is the topic of the essay discussed accurately in the context of the current literature?

Yes

Does the essay contribute to the cultural, historical, social understanding of the field?

Yes

Reviewer Expertise:

I am not a specialist of either Derrida or Heidegger, but rather of Edith Stein's phenomenology. Stein's understanding of the relations between expression, word, concept, idea (essentiality) and essence nevertheless equips one well to understand what is at stake in translation. I have also published several translations and work in several languages.

I confirm that I have read this submission and believe that I have an appropriate level of expertise to confirm that it is of an acceptable scientific standard.

# Reviewer report: Reviewer response for version 1

**DOI:** 10.21956/openreseurope.18472.r38218

**Published:** 2024-04-13

**Authors:** Mette Lebech

**Affiliations:** 1 Maynooth University, Maynooth, County Kildare, Ireland

This paper is about translation. It has a salutary focus on an actual practice, from which many people earn their living and upon which many more are dependent for doing their jobs well. That translation is possible is in consequence not lost from view, and is even seen as a chance for philosophy, a ‘starting point’, maybe, at any rate something that helps to ‘manifest the presuppositions on which’ philosophical texts rests (2, e.)). Translation can thus help clarify what is being said in a text, as much as it can obscure it. One could be tempted to say – keeping the job of the translator in mind – that a good translation is one that clarifies what is being said and a bad one, one that obscures it. Translation can thus ‘test’ a text for its meaning, much like holding up a drawing to a mirror can ‘test’ its composition. This is true even as there are texts part of the meaning of which is that they are obscure, since even that can be well translated. Likening the translation to a ‘mirror’ is an analogy present in a heading in the article even if it is left unexplored. Such exploration may indeed be for another piece of writing.

 The general tenor of the article is thus helpful and sobering. My critical comments are all suggestions for linguistic improvement or clarification. They are all formulated as corrections but should be taken or left according to whether they seem insightful or not.

 Abstract: Replace ‘a “makeshift” or as a shipwreck’ with ‘makeshift or as wandering’.

 P. 2, col. 1, para 2, line 7-6 from bottom: replace ‘be symptoms of’ with ‘characterize’ and delete ‘the concept of’. Introduce a full stop after ‘translation’ in line 6 and reformulate the remainder of the sentence, which is unclear.

 “, line 4 from bottom: it is grammatically unclear what ‘the remodelling’ is a remodelling of. According to its meaning a remodelling presupposes something it **re-**models. To the extent this is intentionally a-grammatical, this should be noted to help the reader.

 P. 3, col. 1, para 4, line 5: replace ‘the human being’ with ‘human beings’ (to align with ‘their’).

 “, line 2 from bottom: replace ‘disqualifying’ with ‘a disqualification'.

 “, last line: delete ‘for translation’.

 P. 3, col. 2, para 1, line 3: replace ‘oneself’ with ‘us’.

 “, line 4: replace ‘of origin’ with ‘original’.

 “, line 2 from bottom: might ‘most distinctive aspect in our sights’ be replaced with ‘ownmost perspective’?

 P. 3, col. 2, para 5: It seems that Derrida is, rather than placing the notion within a ‘family’ (which would make one think of etymology), using these terms as metaphors.

 P. 4, col. 1, para 3, line 4, replace ‘the’ with ‘his’.

 P. 5, col. 1, para 2, line 8-7 from bottom: one does not idiomatically coin concepts, but phrases, terms, or words. Can ‘concept’ here be replaced with ‘terms’?

 “, para 3, line 3: insert ‘’’ after ‘makeshift’.

 “, col. 2, para 1, last (part-)sentence: can it be made a whole sentence? Otherwise, it is difficult to find meaning in the following quotation, supposedly finishing the sentence.

 P. 6, col. 1, para 2, line 14: ‘afterlife’ is not a good translation of ‘Überleben’. ‘Survival’ would be better.

 “, last para, line 4: replace ‘ *the* beauty’ with ‘ *the* beautiful’.

 “, line 5: delete ‘and’ and ‘from it’.

 “, col. 2, para 1, line 4 from bottom: lost by what activity?

 “, para 2, line 6: insert ‘it’ after ‘which’.

 “, line 3 from bottom: replace ‘is’ with ‘to’.

 P. 7, second last line: should ‘word’ be ‘world’?

Is the argument persuasive and supported by appropriate evidence?

Yes

Is the work clearly and cogently presented?

Yes

Is the topic of the essay discussed accurately in the context of the current literature?

Yes

Does the essay contribute to the cultural, historical, social understanding of the field?

Yes

Reviewer Expertise:

I am not a specialist of either Derrida or Heidegger, but rather of Edith Stein's phenomenology. Stein's understanding of the relations between expression, word, concept, idea (essentiality) and essence nevertheless equips one well to understand what is at stake in translation. I have also published several translations and work in several languages.

I confirm that I have read this submission and believe that I have an appropriate level of expertise to confirm that it is of an acceptable scientific standard, however I have significant reservations, as outlined above.

# Response

**Published:** 2024-04-14

**Authors:** Elena Nardelli

Thank you very much for your careful review.

# Reviewer report: Reviewer response for version 1

**DOI:** 10.21956/openreseurope.18472.r38222

**Published:** 2024-03-06

**Authors:** Aaron Turner

**Affiliations:** 1 Knapp Foundation, London, UK

This is an incredibly insightful and detailed analysis of the 'ethics' of translation through the lens of two of the most significant philosophers of the 20th Century, Martin Heidegger and Jacques Derrida. The author demonstrates a comprehensive understanding of these thinkers and their key works, which is especially impressive in the case of Heidegger, whose corpus is vast and his comments on the issues surrounding translation are, as the author notes, are scattered and unsystematic. The main strength of the paper is that it is clearly not a mere recapitulation of the two philosophers' views on translation contrasted against each other but a very thoughtful consideration of the philosophy of translation wherein the author's own voice is clearly heard. 

 The problem of the philosophy of translation is too often overlooked in numerous disciplines these days, not least in classical studies, in which Heidegger had a considerable stake. Heidegger has been frequently criticised for his imaginative and ambitious translation of Greek texts, which critics suggest reflect not the original author but Heidegger himself. In this article, the author does tremendously admirable work in elucidating Heidegger's justifications for his translations which classicists seldom acknowledge. For this reason, the article will serve as an important contribution to modern scholarship and lay the groundwork for further elaborations of Heidegger's (and Derrida's) philosophy of translation. 

 Perhaps the author could have acknowledged some of these issues in the introductory sections of the paper, especially as these issues have been a source of intense criticism of Heidegger in particular. Perhaps, as well, the author could have expanded on Heidegger's work on Heraclitus and the interpretation of *logos* throughout the 1930s and early 1940s, which, together with his work on Hölderlin, transformed Heidegger's understanding of language and formed the basis of much of what came later. However, such expansions and elaborations would perhaps detract from the concise and focused argument that carries through the chapter and as such, while they would be interesting to consider, I would not recommend for them to be included.

 Ultimately, this article is a significant and worthy contribution to the discourse surrounding the problem of the 'ethics' of translation.

Is the argument persuasive and supported by appropriate evidence?

Yes

Is the work clearly and cogently presented?

Yes

Is the topic of the essay discussed accurately in the context of the current literature?

Yes

Does the essay contribute to the cultural, historical, social understanding of the field?

Yes

Reviewer Expertise:

I work primarily in the areas of classics, philosophy, and history. I have an excellent understanding of Heidegger and a good awareness of Derrida's work. My work covers translations of ancient Greek and German so I'm well acquainted with the practical and philosophical issues of translation, which is precisely the concern of the article.

I confirm that I have read this submission and believe that I have an appropriate level of expertise to confirm that it is of an acceptable scientific standard.

# Response

**Published:** 2024-05-06

**Authors:** Elena Nardelli

Thank you very much for your careful reading.
